# Giant Splenic Artery Aneurysm: Case Report

**DOI:** 10.5402/2011/383450

**Published:** 2011-04-27

**Authors:** Sadaf Ali, Vibha Verma, Sastry R, Imtiaz Wani

**Affiliations:** ^1^Department of Surgical Gastroenterology, SKIMS, Srinagar, Kashmir, India; ^2^Department of Surgical Gastroenterology, Nizam's Institute of Medical Sciences, Hydrebad, India

## Abstract

Splenic artery aneurysm is the third most common location of intra-abdominal aneurysms. Giant splenic artery aneurysm is rarely seen and is at a high risk of rupture. Location and size of the splenic artery aneurysm determine the likelihood of rupture. A case of giant splenic artery aneurysm in a 35-year-old woman is reported. She presented with upper gastrointestinal bleeding. She had splenomegaly and extrahepatic hepatic portal hypertension. Angiography confirmed a giant splenic artery aneurysm measuring 8 × 10 centimeters, located in middle and distal two-thirds of the splenic artery. Surgical treatment in the form of in toto excision of aneurysm with splenectomy and devascularization was performed.

## 1. Introduction

Splenic artery aneurysm accounts for approximately 60% of all visceral arterial aneurysms [1]. Splenic artery aneurysm is the third most common location of intraabdominal aneurysms following the abdominal aorta and iliac arteries [2]. Giant splenic artery aneurysms are rarely seen. Clinical presentation of a splenic artery aneurysm is variable with most patients being asymptomatic. The aneurysm is found incidentally on imaging studies. Computed tomography and magnetic resonance imaging demonstrate morphology and location of the aneurysm. Color Doppler ultrasound can be diagnostic. Surgical treatment options are aneurysm resection or simple ligation and splenectomy.

## 2. Case Report

**Figure 1:**
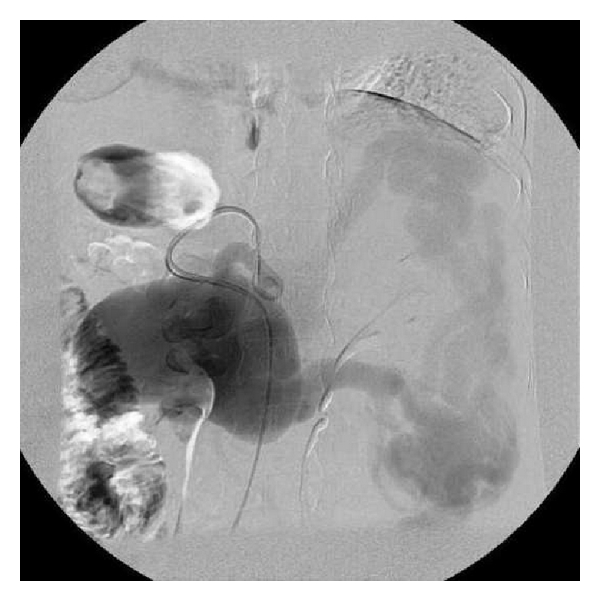
Angiography demonstrates aneurysm in the splenic artery with dilated tortuous vessel around suggestive of portal hypertension.

**Figure 2:**
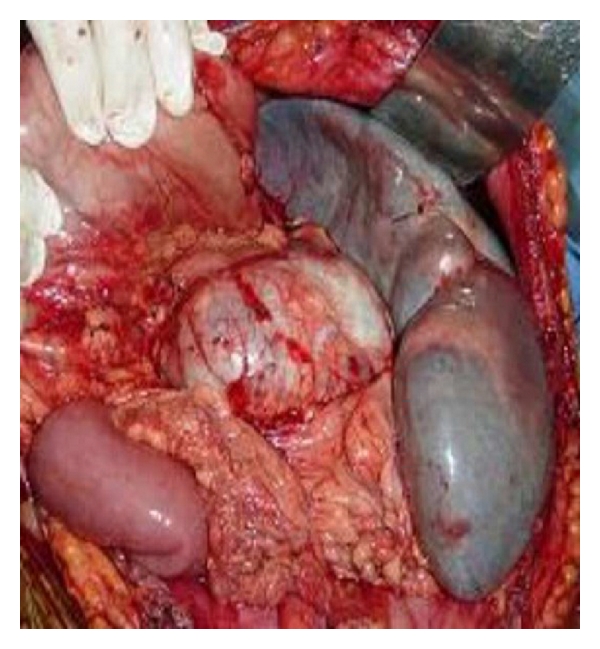
Showing giant aneurysm of splenic artery with splenomegaly.

**Figure 3:**
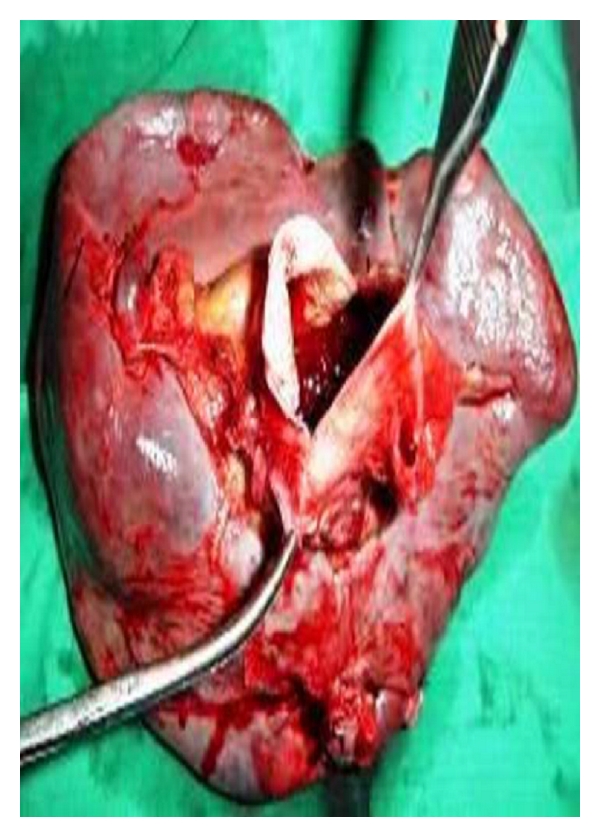
Showing lumen of giant splenic artery aneurysm.

A 35-year-old woman presented with three episodes of passage of black tarry stools. She was para 2 and had last childbirth 2 years back. There was no history suggestive of any systemic infections, trauma, family history of aneurysm, and connective tissue disorders. The patient had an anxious look, pallor, pulse rate of 120/min. and B.P of 90/70 mm Hg. An emergency upper gastrointestinal endoscopy was done which revealed grade II-III oesophageal varices for which sclerotherapy was done. Systemic examination was normal. Abdominal examination revealed splenomegaly. Hemoglobin was 9 gm/dL, and liver function tests were normal. Ultrasonography of abdomen showed massive splenomegaly and a cyst in relation to splenic artery. Doppler study was suggestive of extrahepatic portal vein obstruction (EHPVO) with splenic artery aneurysm and multiple perisplenic, gastric, and perioesophageal collaterals and documenting portal cavernoma with hepatofugal flow (Figure 1). On digital subtraction angiography a selective angiogram confirmed a huge saccular aneurysm arising from splenic artery. Patient was offered endovascular intervention but refused. On laparotomy a normal appearing liver, no ascites, and a 8 × 10 centimeters aneurysm arising from splenic artery, involving middle and distal two-thirds of an artery and an enlarged spleen were observed (Figure 2). Multiple collaterals, significantly on the left sided, were present. Aneurysmectomy with splenectomy was done (Figure 3). Splenic aneurysm biopsy revealed true aneurysm, splenic biopsy showed congestion with small areas of infarction, and liver biopsy was normal. This patient had an uneventful postoperative period and was discharged on the 10th postoperative day.

## 3. Discussion

Splenic artery aneurysms larger than 3 centimeters are rare, but giant aneurysms measuring 10 cm or more have been reported [3]. Splenic artery aneurysm has a female predominance, can be diagnosed at any age, but is more commonly seen in the fifth and sixth decades with a mean age of presentation of 52 years [4]. These are generally located in the middle and distal segment of the artery and are mostly of saccular form. Specific causes of splenic artery aneurysms remain unknown, although suspected etiological factors are thought to be atherosclerosis, focal arterial inflammation, pancreatitis, hypersplenism, portal hypertension, trauma, and hormonal and hemodynamic changes due to pregnancy, liver transplant, and splenomegaly [5]. These aneurysms can be asymptomatic and in up to 20% may present with epigastric or left upper quadrant abdominal pain. An aneurysm of 5 cm has the risk of rupture and bleeding. Occasionally, the aneurysm can erode into an adjacent viscus or into the pancreatic duct and presents as gastrointestinal hemorrhage.

Generally they are incidentally diagnosed on autopsies or abdominal radiographic examinations. Contrast-enhanced CT scans and CT angiogram are important in the evaluation of this condition. Doppler ultrasound represents a fast and noninvasive strategy and is considered modality very reliable in diagnosis and has great potential as follow-up tools. Once the diagnosis has been confirmed, a therapeutic strategy needs to be determined. Treatment is recommended for all patients with symptomatic aneurysms, those with aneurysm larger than 2 centimeters in a diameter, growing aneurysms, and for all pregnant women and women of childbearing age who may subsequently become pregnant. The choice is governed by the condition of the patient, the exact morphology of the aneurysm, and the availability of the endovascular procedure facility. Endovascular procedure is considered as a first choice of treatment for splenic artery aneurysm. Open surgery is reserved mostly for the treatment of complications or if the endovascular procedures fail or are lack of availability of endovascular procedures or allergic to contrast medium. Surgical procedure for splenic artery aneurysm may involve splenic artery ligation, aneurysmectomy, or splenectomy is the option. If the aneurysm is located in the distal third of the artery and the aneurysm has to be resected, the patient may ultimately require a splenectomy [6]. If the aneurysm is located in the proximal third of the artery, splenic conservation should be attempted.

## 4. Conclusion

Giant splenic artery aneurysm is rare, and pregnancy could be causative.

## References

[1] Feo CF, Scanu AM, Fancellu A, Costantino S (2004). Visceral aneurysm and vascular anomaly involving the splenic artery. *Digestive Diseases and Sciences*.

[2] Trastek VF, Pairolero PC, Joyce JW (1982). Splenic artery aneurysms. *Surgery*.

[3] Huang IH, Zuckerman DA, Matthews JB (2004). Occlusion of a giant splenic artery pseudoaneurysm with percutaneous thrombin-collagen injection. *Journal of Vascular Surgery*.

[4] Dave SP, Reis ED, Hossain A, Taub PJ, Kerstein MD, Hollier LH (2000). Splenic artery aneurysm in the 1990s. *Annals of Vascular Surgery*.

[5] Abbas MA, Stone WM, Fowl RJ (2002). Splenic artery aneurysms: two decades experience at Mayo Clinic. *Annals of Vascular Surgery*.

[6] De Perrot M, Bühler L, Deléaval J, Borisch B, Mentha G, Morel P (1998). Management of true aneurysms of the splenic artery. *American Journal of Surgery*.

